# Mesoporous Carbon Fibers with Tunable Mesoporosity for Electrode Materials in Energy Devices

**DOI:** 10.3390/molecules26030724

**Published:** 2021-01-30

**Authors:** Ting-Wei Huang, Mayumi Nagayama, Junko Matsuda, Kazunari Sasaki, Akari Hayashi

**Affiliations:** 1 Department of Hydrogen Energy Systems, Kyushu University, 744 Motooka, Nishi-ku, Fukuoka 819-0395, Japan; ck12332@gmail.com (T.-W.H.); sasaki@mech.kyushu-u.ac.jp (K.S.); 2 COI-C2RSC, Kyushu University, 744 Motooka, Nishi-ku, Fukuoka 819-0395, Japan; nagayama.mayumi.630@m.kyushu-u.ac.jp; 3 International Research Center for Hydrogen Energy, Kyushu University, 744 Motooka, Nishi-ku, Fukuoka 819-0395, Japan; matsuda.junko.925@m.kyushu-u.ac.jp; 4 NEXT-FC, Kyushu University, 744 Motooka, Nishi-ku, Fukuoka 819-0395, Japan; 5 Q-PIT, Kyushu University, 744 Motooka, Nishi-ku, Fukuoka 819-0395, Japan

**Keywords:** carbon, fiber, porous, energy device

## Abstract

To improve the properties of mesoporous carbon (MC), used as a catalyst support within electrodes, MC fibers (MCFs) were successfully synthesized by combining organic–organic self-assembly and electrospinning deposition and optimizing heat treatment conditions. The pore structure was controlled by varying the experimental conditions. Among MCFs, MCF-A, which was made in the most acidic condition, resulted in the largest pore diameter (4–5 nm), and the porous structure and carbonization degree were further optimized by adjusting heat treatment conditions. Then, since the fiber structure is expected to have an advantage when MCFs are applied to devices, MCF-A layers were prepared by spray printing. For the resistance to compression, MCF-A layers showed higher resistance (5.5% change in thickness) than the bulk MC layer (12.8% change in thickness). The through-plane resistance was lower when the fiber structure remained more within the thin layer, for example, +8 mΩ for 450 rpm milled MCF-A and +12 mΩ for 800 rpm milled MCF-A against the gas diffusion layer (GDL) 25BC carbon paper without a carbon layer coating. The additional advantages of MCF-A compared with bulk MC demonstrate that MCF-A has the potential to be used as a catalyst support within electrodes in energy devices.

## 1. Introduction

As prominent supports for precious metal catalysts, carbon materials have various advantages. In general, carbon materials can practically be used in both acidic and basic environments, and their large surface area leads to good dispersion of precious metal particles to improve metal utilization [1,2,3]. Further, carbon does not exhibit solid acid–base properties for metal anchoring, so its valence state is not affected [4]. Furthermore, for recovering only metal out of metal particles deposited on carbon materials, carbon is easily removed through direct pyrolysis, which is another good feature as a support in terms of recycling metal. When carbon materials are partially or fully graphitized, they increase electrical conductivity and thermal conductivity; both are essential properties for electrodes of electrochemical devices. Many different carbon substrates, such as activated carbon [5] and carbon black [6], have been utilized as metal supports in the past decades.

Beyond common manufactured carbon materials, the development of novel nanostructured carbon materials with a porous structure has received more attention in energy-related fields because of their enlarged surface area. The resulting large surface area can provide not only large electrical double-layer capacitance [7], but also better dispersion of metal catalysts [8]. Such nanostructured carbon materials can be divided into different groups based on their pore size, such as microporous (pore size <2 nm), mesoporous (2–50 nm), and macroporous (>50 nm) carbon materials.

Among the different types of porous carbon materials, mesoporous carbon (MC) with relatively large mesopores over 5 nm was developed by several researchers in the early 2000s [9,10,11]. Such MC has great potential in electrochemical fields since the mass transfer of reactants and products for chemical reactions is not limited in the mesopore system, while such mass transfer is limited in the microspore system [12]. Additionally, the curvature within mesopores is known to reduce the mobility of metal particles and then improve the durability [13].

Our group has been working on an electrode using MC [14,15,16,17,18]. We have found that when MC is used in the electrodes of devices, the disordered structure at the micrometer scale rather brings forth some mass transfer and electrical conductivity issues. Therefore, controlling the micrometer-scale structure of electrode materials seems to be an effective strategy to improve the electrochemical performance, as reported in the supercapacitor [19,20] and Li-S battery [21] research areas. The conductive path formed by fiber-like materials, such as carbon nanotubes (CNTs) and carbon nanofibers (CNFs), can provide better electronic conductivity to enhance the performance, although conventional CNFs or CNTs without surface treatments are usually poor in surface area. For example, CNFs showed poor Li particle dispersion without anchoring points of Ag nanoparticles [22]. Therefore, although we are similarly interested in introducing the fiber structure, we are particularly focused on high surface areas with mesopores, where small nanoparticles of catalysts can be impregnated. The electrospinning deposition (ESD) technique is desirable in our case since the precursor of our MC material, known as resorcinol-based resin, is often used as a precursor material of ESD-made carbon fibers [23].

Regarding other work on MC fibers (MCFs) using ESD, Wang et al. [24] provided a way to make carbon fibers with 6 nm mesopores using a solution containing carbon and silica precursors with a removal procedure of a silica hard template by HF. Ghosh et al. [25] reported a similar way of using SiO_2_ as a pore inducer and removing it by HF, and they obtained larger pores with diameters of 20–40 nm. In both cases, an extra step of removing a silica hard template by HF is required. Ma et al. [26] provided a synthesis process without extra acid etching to make MCFs by using additive KOH in their precursor resin and changing its content, leading to easy tuning of the pore volume. Wang et al. [27] reported porous carbon nanofibers prepared from electrospun PAN/novolac only through a N_2_ calcination process. In both cases, additional acid etching was not necessary, but the diameter of the mesopores was small (e.g., below 2 nm), which is not suitable for further metal impregnation into the mesopores. Ozaki et al. [28] instead produced fibers through a small nozzle attached to a melting pot containing the polymer blend with different thermal stabilities, resulting in the largest pore size of 8 nm. Nevertheless, mesoporosity was not easily tunable.

The aim of this study was to develop MCFs simply through ESD and heating processes. We combined our original MC synthesis and ESD. The mixture of carbon precursors, Pluronic^®^ F127, and additional linear polymers was spun and further treated by N_2_ calcination to obtain MCFs. Control of the mesopore size and volume was attempted. Besides common material characterization, by making thin layers of MCFs, the resistance to compression and the through-plane electric resistance were evaluated in anticipation of applications for catalyst supports within electrodes of energy devices.

## 2. Results and Discussion

### 2.1. Characterization of the Porous Structure of MCFs

**Figure 1:**
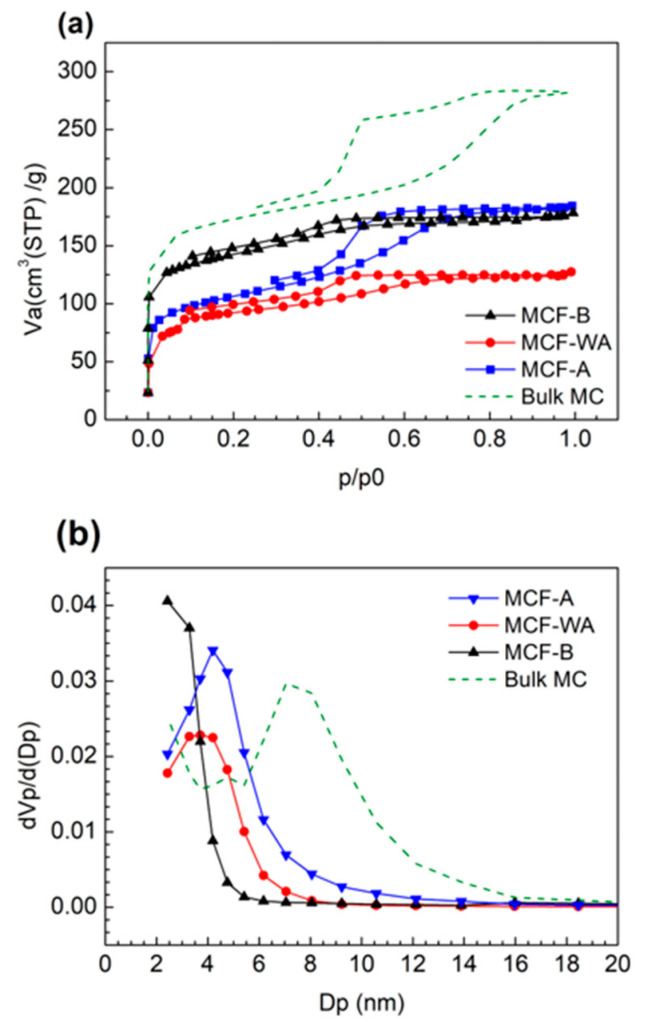
Nitrogen sorption isotherms (**a**) and the corresponding pore distributions, (**b**) of different MCF samples and bulk MC as a reference sample.

The N_2_ adsorption/desorption measurements of MCFs made from precursor solutions with different pH values—MCF-A (pH = 3.8), MCF-WA (pH = 4.6), and MCF-B (pH = 7.6)—were evaluated, and their N_2_ sorption isotherms and corresponding pore distributions were compared (Figure 1). All types of MCFs showed the type IV isotherm, indicating a mesoporous structure. The main pore diameters were 4–5, 3–4, and 2–3 nm for MCF-A, MCF-WA, and MCF-B, respectively, and they decreased along with increasing pH values of the precursor solutions. Regarding Brunauer-Emmett-Teller (BET) surface area, MCF-A, MCF-WA, and MCF-B revealed 370, 340, and 670 m^2^/g, respectively. The increase in BET surface area for MCF-B was mostly due to the increase in micropores. These properties are summarized in Table 1, with bulk MC as a reference sample.

According to Tanaka et al. [29], the size of mesopores is mainly determined by the size of surfactant micelles. Since the bonding between surfactant micelles and carbon precursors mainly consists of hydrogen bonding [30], a decrease in the number of hydrogen bonds in a basic condition is expected, which leads to crushing of the micelles during ESD and then results in smaller pores.

For our purpose, the MCF was designed to be utilized as a supporting material of catalytic metal particles with a dimeter of 2–3 nm; the larger the mesopore size, the better the reactant transportation. Since a further increase in acidity of MCF precursor solutions increases the instability of the ESD process because of partially hydrolyzed polyvinyl alcohol (PVA) [31], MCF-A, which had the largest pore diameter, was chosen for further characterization in this study.

### 2.2. Optimization of the Heat Treatment Condition of MCF-A

**Figure 2:**
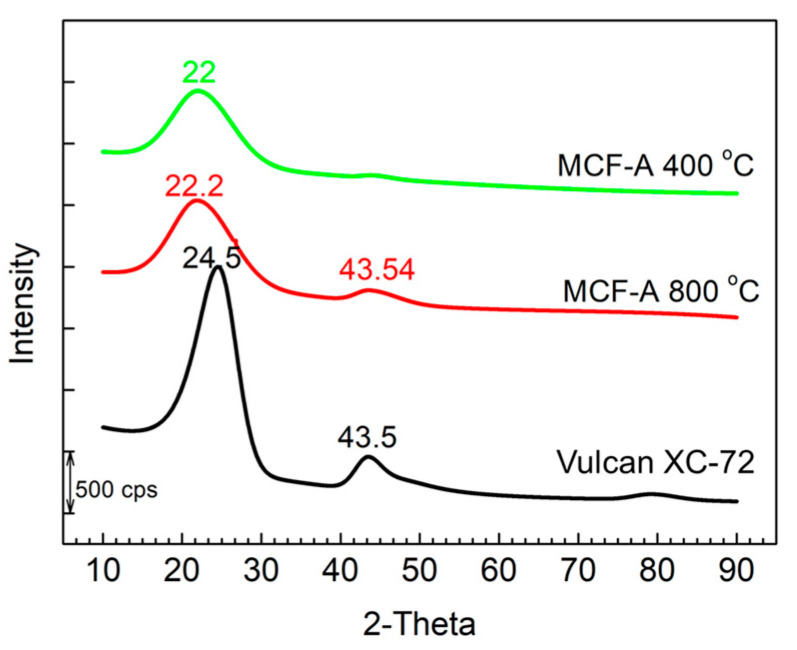
XRD patterns of MCF-A under each calcination step and Vulcan XC-72 for comparison.

All of the different types of MCFs were first treated with sequential heating steps of 400 + 800 °C based on our previous synthesis of bulk MC [14]. Using MCF-A, the change in crystallinity under the each calcination step was further evaluated by XRD, as shown in Figure 2. Here, XRD patterns of Vulcan^®^ XC-72 were also added to the figure for comparison. The relatively low peak intensity and high peak broadening compared with graphite are typical features of the amorphous structure of carbon samples. Vulcan^®^ XC-72 only showed two prominent peaks at 24.5° (major) and 43.5°, indicating C (1 1 1) and C (2 0 0), respectively. The sample treated at 400 °C showed only one major peak located at 22.0°, which was a shifted C (1 1 1) peak. With additional N_2_ calcination at 800 °C, besides the major peak of the MCF-A precursor, a secondary peak began to appear at 43.5°, indicating that the MCF-A precursor turned into MCF-A with a graphite-like structure.

To further understand the degree of carbonization, the heat treatment condition of the second step was slightly modified. MCF-A with steps of 400 °C + 700 °C, 400 °C + 700 °C + 800 °C, and 400 °C + 700 °C + 800 °C + 900 °C were prepared, which is explained in Section 3.4 in detail. Carbon-hydrogen-nitrogen (CHN) analysis for them was performed, and their atomic percent of H and C are listed in Table 2. As a result, the atomic percent of H decreased along with the increase in the calcination temperature. The atomic percent of C kept increasing until the calcination temperature reached 800 °C. Since a further increase in the carbon atomic ratio was not seen at 900 °C, heat treatment up to 800 °C was chosen as a carbonization condition in this study to synthesize MCF-A.

**Figure 3:**
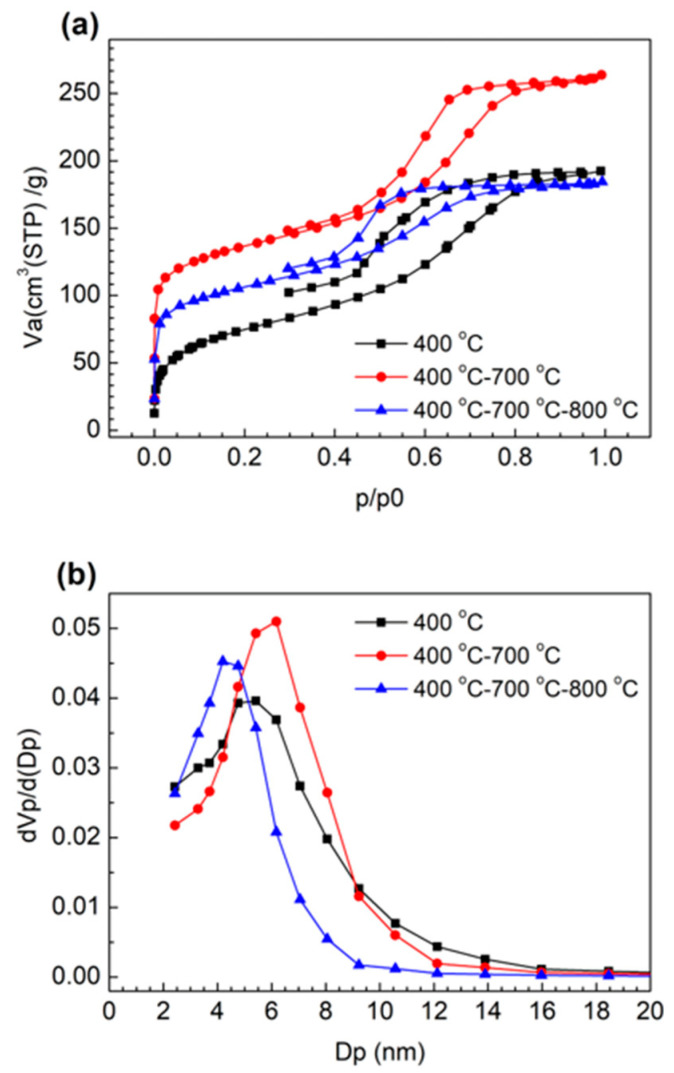
Nitrogen sorption isotherms (**a**) and the corresponding pore distributions (**b**) of MCF-A treated with different temperature.

Besides MCF-A with 400 °C + 700 °C + 800 °C + 900 °C treatment, which already showed no further change in C content, N_2_ adsorption/desorption isotherms and corresponding pore distributions of MCF-A precursors treated at 400 °C, 400 °C + 700 °C, and 400 °C + 700 °C + 800 °C were further analyzed, as shown in Figure 3. Although the MCF-A precursor without any heat treatment showed almost no N_2_ adsorption (not shown here), the MCF-A precursor treated at 400 °C started adsorbing N_2_. The BET specific surface area and main pore diameter were 240 m^2^/g and 5–6 nm, respectively. With the heat treatment with additional 700 °C calcination, the BET surface area and main pore diameter further increased to 490 m^2^/g and 6–7 nm, respectively.

On the other hand, with further 800 °C calcination, the BET surface area and main pore diameter decreased to 380 m^2^/g and 4–5 nm, respectively. This was probably due to shrinkage by further removal of H and O atoms, which was also partly found in the above CHN element analysis. However, compared with MCF-A obtained by direct heating from 400 to 800 °C, as shown in Figure 1, even though the main pore diameter did not change much, the pore volume, which can roughly be compared from the area under the curve, was found to decrease when heating to 800 °C directly from 400 °C.

Although the pore diameter was larger with the heat treatment up to 700 °C, the higher carbonization degree obtained from further heating to 800 °C is rather important as an electrocatalyst support for our study, so the heat treatment up to 800 °C was chosen in this study. Based on the results above, carbonization of precursor fibers was optimized as follows: the first step was heating to 400 °C with a rate of 2 °C/min and keeping the temperature at 400 °C for 3 h. The second step was heating to 700 °C with a rate of 2 °C/min and keeping the temperature at 700 °C for 3 h. The final step was heating to 800 °C with a rate of 2 °C/min and keeping the temperature at 800 °C for 6 h to obtain MCF-A.

### 2.3. Characterization of Morphology of MCF-A

**Figure 4:**
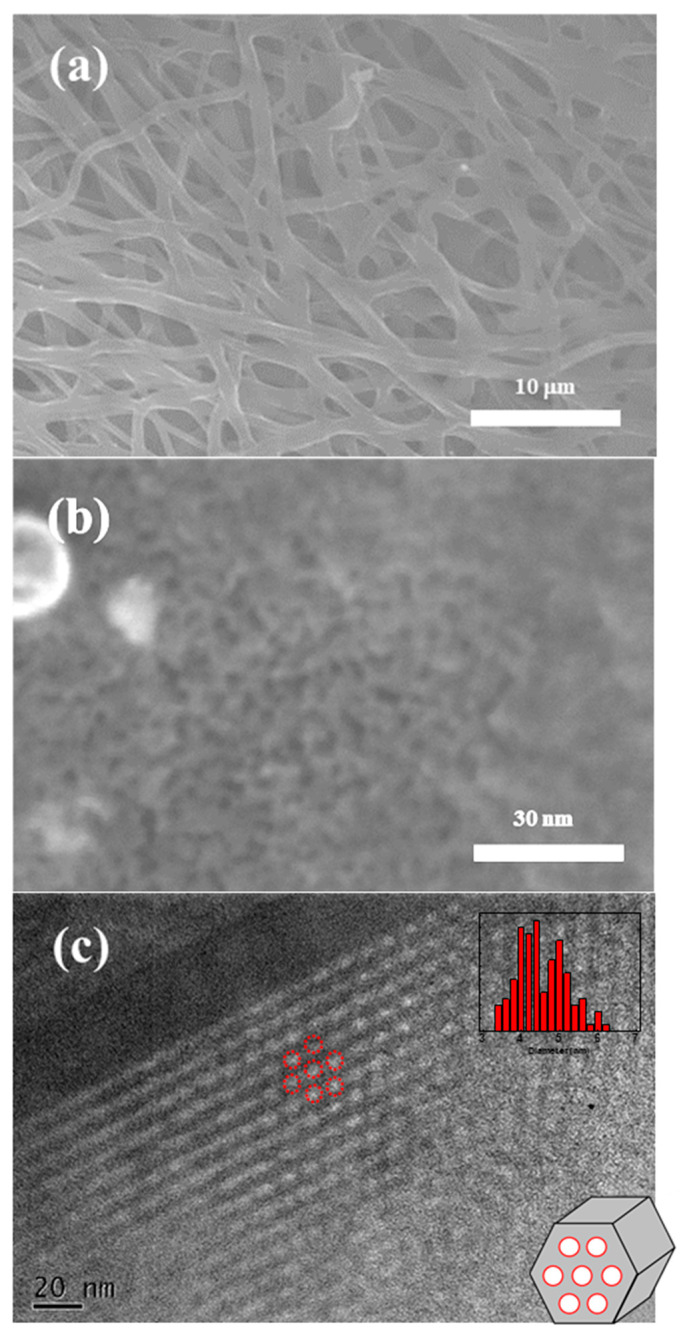
SEM images with low magnification (**a**) and high magnification (**b**), and a TEM image with pore size distribution (**c**) of MCF-A after 800 °C calcination.

The morphology of MCF-A was evaluated by SEM and TEM and is shown in Figure 4. As shown in Figure 4a, the diameter of the fibers was found to be within the range of 500 nm to 2 μm. Based on the high magnification SEM image of Figure 4b, mesopores with a diameter roughly around 4–5 nm can clearly be observed on the surface of the fibers with a random arrangement. However, as seen in Figure 4c, hexagonally ordered mesopores can be confirmed in the TEM image. Moreover, the average pore size was found to 4.6 nm through the image processing, explained in Section 3.5. The resulting pore diameter is well matched to that obtained from N_2_ adsorption measurements. There are also micropores, which are probably made during the heat treatment, contributing to the increase in the specific surface area.

### 2.4. Electrochemical Characterization of MCF-A Powder

**Figure 5:**
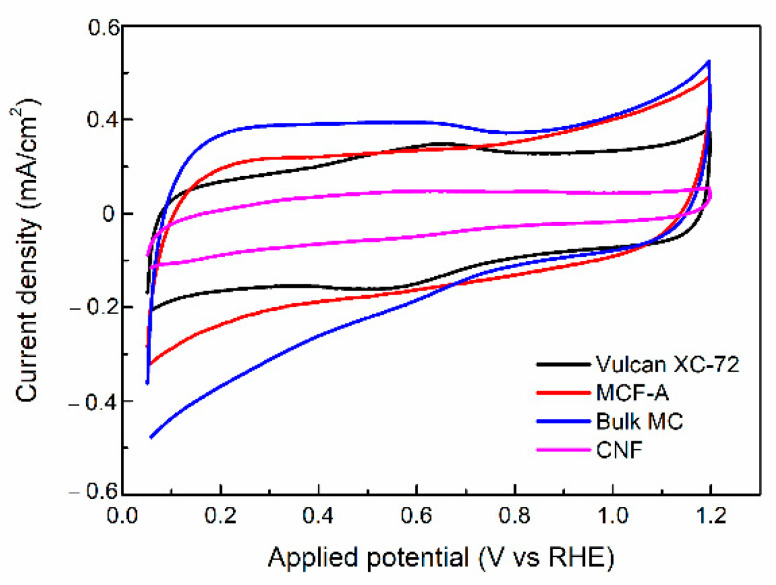
Cyclic voltammograms (CVs) of MCF-A, bulk MC, Vulcan^®^ XC-72, and carbon nanofibers (CNFs) obtained in N_2_-saturated HClO_4_.

Fundamental electrochemical properties of MCF-A and other reference carbon materials, such as bulk MC, carbon black Vulcan^®^ XC-72, and CNF, were evaluated. First, cyclic voltammograms (CVs) were obtained within the voltage range from 0.05 to 1.15 V (vs reversible hydrogen electrode (RHE)) with a scan rate of 50 mV/s, as shown in Figure 5. All of the CVs show a slightly distorted rectangular shape. Vulcan^®^ XC-72 shows a broad anodic peak around 0.7 V, resulting from the adsorption of O atoms on the carbon surface, and a corresponding cathodic peak around 0.6 V. Bulk MC also shows a similar current response even though it is smaller. According to the literature [32], carbon materials derived from the phenolic resin have oxygen-containing functional groups on their surface and show the similar CVs.

The specific capacitances (C_p_) of MCF-A, bulk MC, Vulcan^®^ XC-72, and CNF were 56.5, 62.4, 47.9, and 15.6 F/g, respectively, based on the method of calculation described in the experimental section. The order of C_p_ within the four samples was found to be reasonable based on their BET surface area obtained from N_2_ sorption measurements: 380, 600, 220, and 130 m^2^/g for MCF-A, MC, Vulcan^®^ XC-72, and CNF, respectively. The reason for the lowest C_p_ and BET surface area of CNF might be due to the defect-less structure of the particular sample in this study made from the chemical vapor deposition process.

**Figure 6:**
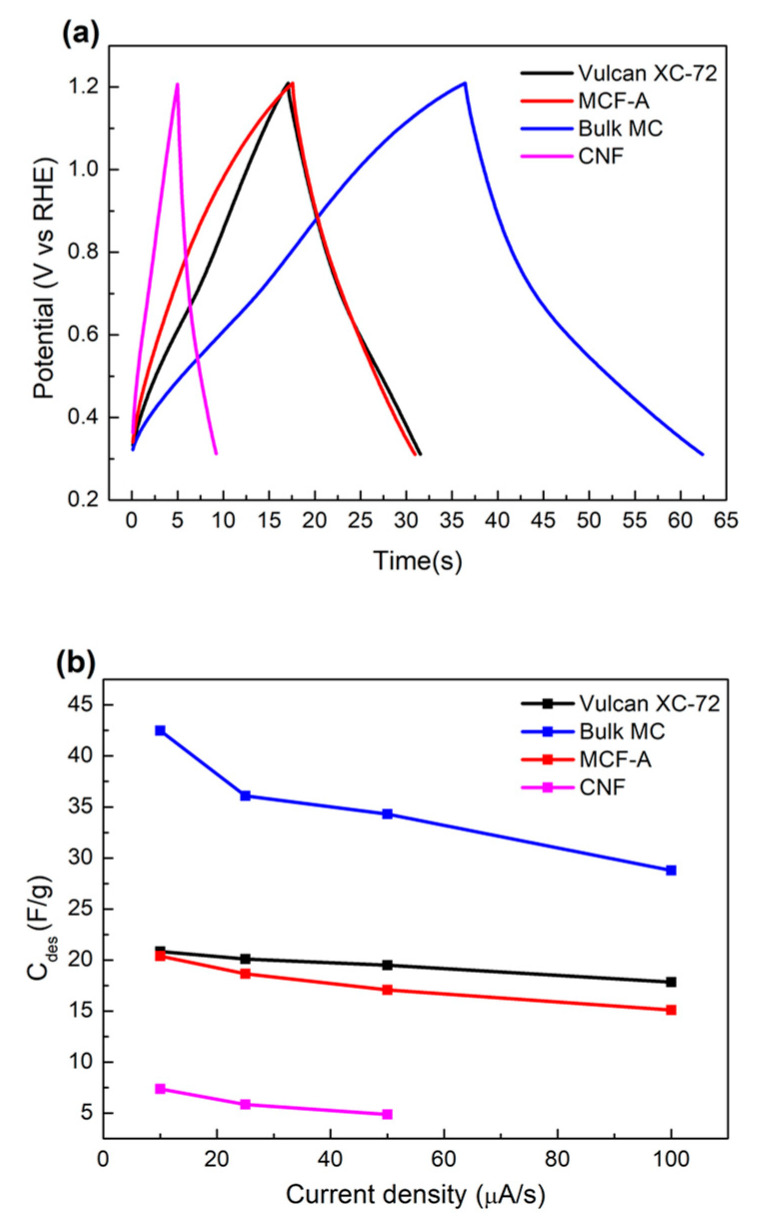
Charge and discharge (CDC) responses of voltage under application of 25 μA (**a**) and discharge-specific capacitance under different discharging current densities (**b**) for different carbon materials obtained in N_2_-saturated HClO_4_.

**Figure 7:**
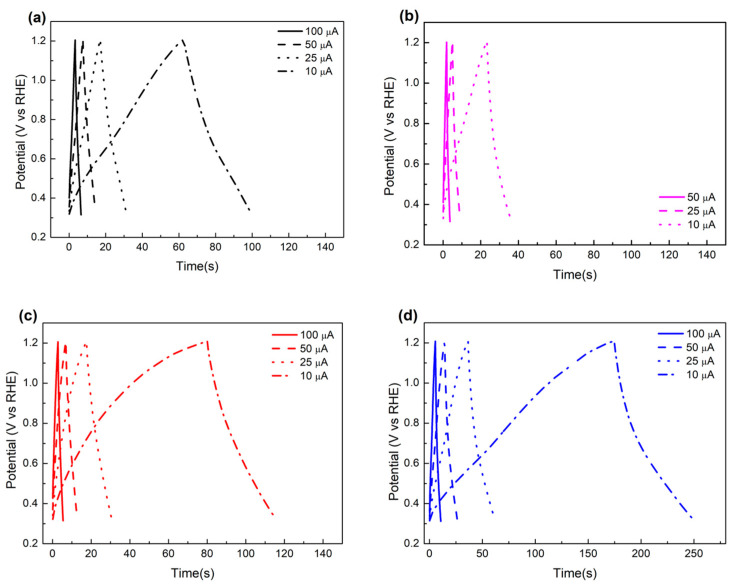
CDC responses of voltage of Vulcan XC-72 (**a**), CNF (**b**), MCF-A (**c**), and bulk MC (**d**) under different charging currents, where the X-axis for (**a**), (**b**), and (**c**) is 0–140 s, and the X-axis for (**d**) is 0–275 s, obtained in N_2_-saturated HClO_4_.

Next, charge and discharge (CDC) measurements were performed by applying a charging current of 25 μA for different carbon materials, as shown in Figure 6a. Discharge-specific capacitance (C_des_) calculated in the different charging current is also plotted in Figure 6b. The C_des_ decreased with the increase in the charging current for all samples, which is a common feature of carbon-based materials. CDC voltage response under the different charging currents was further analyzed, and is shown in Figure 7. The hysteresis of voltage in the potential range near the end of a charging/discharging process was found in the MCF-A and MC cases. Since increase in voltage during the CDC process is related to electron transfer resistance and ion diffusion resistance, the resulting hysteresis might be a feature of hydrophobic mesopores. Although the initial pH conditions of the MCF precursor solutions might result in different hydrophilic/hydrophobic natures of mesopores, leading to different capacitor performances [33], no further analysis has been done since CDC measurements were performed for the fundamental electrochemical properties of carbon, and the aim of this study was not to develop supercapacitance, but catalyst supports within electrodes in energy devices.

### 2.5. Characterization of MCF-A Layer

**Figure 8:**
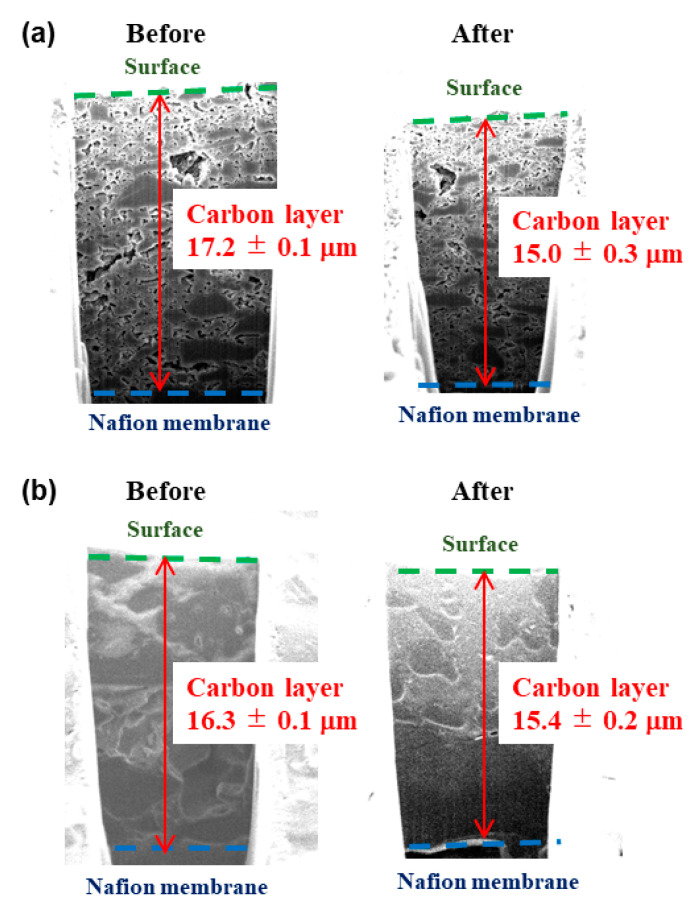
Cross-sectional view of bulk MC layer (**a**) and MCF-A layer (**b**) before and after the compression test. The thickness of each carbon layer is shown with red arrows.

Since the fiber structure is expected to have an advantage when MCF is applied to devices, MCF-A ball-milled at 450 rpm was spray-printed onto a Nafion 212 membrane as a layer, and the resistance to compression of 2 Nm under a cell holder for regular current–voltage performance measurements was evaluated, as explained in the experimental section. Similarly, the bulk MC layer was also prepared and evaluated. Cross sections of the MCF-A layer and bulk MC layer were fabricated and observed using a Focused ion beam (FIB)-SEM technique, and typical images are shown in Figure 8. The thicknesses of the carbon layers are also noted in the figure. According to the average thickness measured before and after compression, the change in the MCF-A layer was about 5.5% (16.3–15.4 μm) and was lower than the change in the bulk MC layer (12.8%; 17.2–15.0 μm).

**Figure 9:**
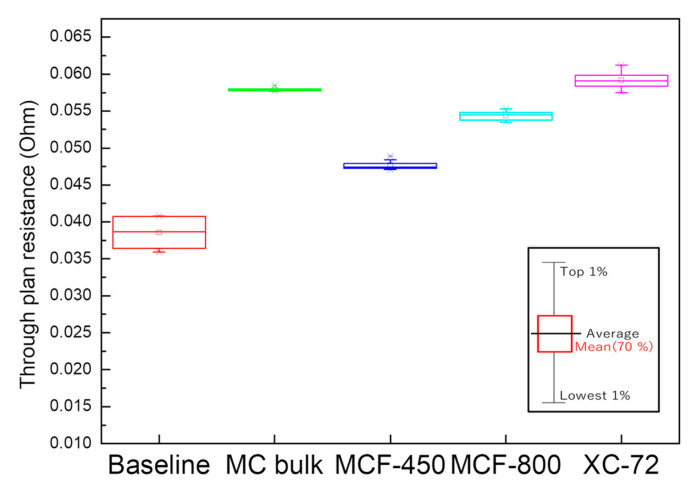
Through-plane electronic resistance of different carbon samples with the inset explaining the meanings of the symbols.

**Figure 10:**
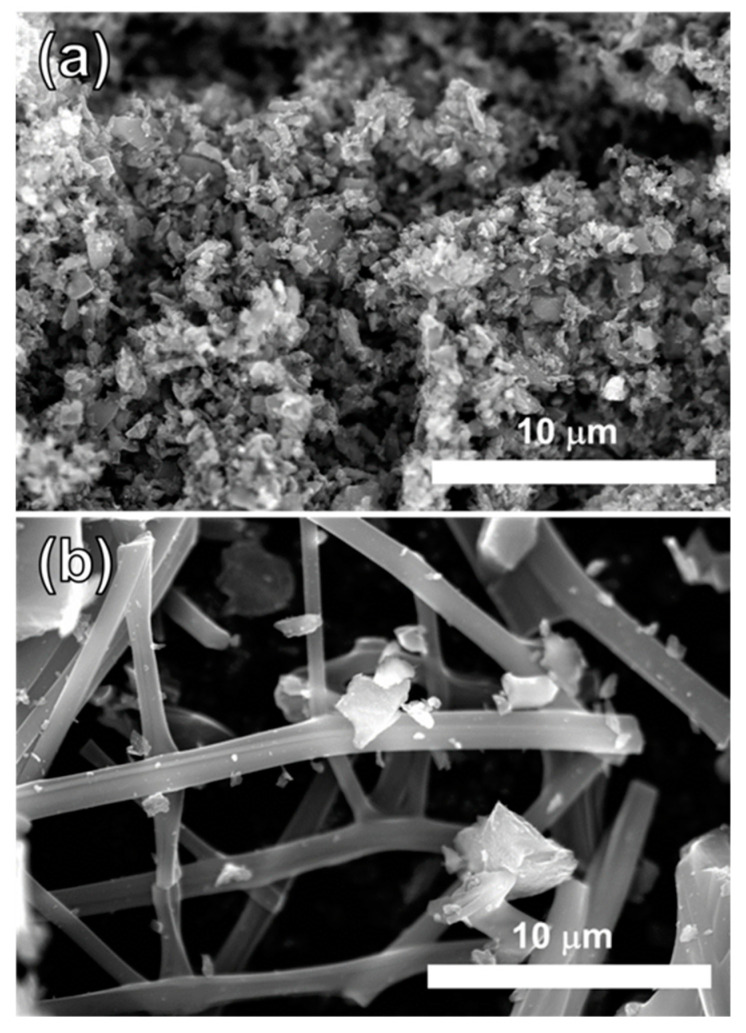
SEM images of MCF-A-800 (**a**) and MCF-A-450 (**b**).

Since decreased resistance with fiber structure is also expected in devices, the through-plane resistance of MCF-A was evaluated by spray printing on GDL 25BC carbon paper as a layer and compared with bulk MC and Vulcan XC-72 (XC-72), as described in Section 3.8. For MCF-A, two samples—MCF-A ball-milled at 450 rpm (MCF-A-450) and MCF-A ball-milled at 800 rpm (MCF-A-800)—were evaluated. The obtained through-plane resistance is shown in Figure 9. Bulk MC and XC-72 were both +20 mΩ higher than the baseline, but MCF-A-800 and MCF-A-450 were +12 mΩ and +8 mΩ, respectively. According the SEM images shown in Figure 10, MCF-A-450 (Figure 10b) was found to have longer fibers than MCF-A-800 (Figure 10a). Therefore, the difference in resistance between MCF-A-450 and MCF-A-800 comes from the difference in the fiber length. When more of the fiber structure remains, the through-plane resistance is lower. Consequently, fiber structure was found to have an advantage in conductivity, especially if the fibers are longer and connected.

## 3. Materials and Methods

### 3.1. Materials

All of the chemicals were used as received without further purification. Resorcinol, phloroglucinol dihydrate, ethanol (99.5 purity), 5 M HCl, triethyl orthoacetate (EOA), formaldehyde solution, 2-propanol, sodium hydroxide (tablet), 20% ammonia solution, and 5% Nafion^®^ dispersion solution (DE520 CS type) were purchased from Wako Pure Chemical Industries Ltd (Osaka, Japan). Mowiol^®^ 40-88, Pluronic^®^ F127, and CNF (100 nm diameter and 10–20 mm length) were purchased from Sigma-Aldrich, Inc. (St. Louis, MO, USA). Hydrophobic carbon paper with polytetrafluoroethylene (EC-TP1-060T), hydrophobic carbon paper with polytetrafluoroethylene with a microporous layer (GDL 25BC), Vulcan XC-72, and Nafion^®^ 212 were obtained from Toray Industires, Inc. (Tokyo, Japan), SGL carbon (Wiesbaden, Germany), Cabot Corp. (Boston, MA, USA), and Dupont (Wilmington, DE, USA), respectively. Milli-Q water was used in all of the experiments.

### 3.2. Preparation of MCF Precursor Solutions for ESD

Concerning ESD, large molecules of linear polymers in addition to the precursor are required in order to shield the precursor solution from high voltages and to prevent the breakage of Pluronic^®^ F127 micelles; so, a template was used for the porous structure in this study. The selection of linear polymers in the ESD process is very important. Based on the study of Stachewicz et al. [34], with a positive voltage being applied, molecules in the precursor solution tend to rearrange in the order of electronegativity. Molecules with higher electronegativity tend to stay in the outer phase and become a shielding layer of fibers during ESD. Then, chain polymers such as PVA [35], polyvinyl butynol [36], or polyvinyl pyrrolidone [37] are suitable additives for ESD of the MCF precursor solution. In this research, Mowiol^®^ 40-88, which is PVA with Mw of 31,000 and degree of hydrolysis of 88%, was used as a linear polymer additive for ESD.

The MCF precursor solution was made as follows: the 13 wt % PVA solution was separately prepared dissolving 2.6 g of PVA into 17.4 g of Milli-Q water under stirring at 85 °C for 4 h and cooled down before use. Phloroglucinol dihydrate (0.64 g), 0.6 g of Pluronic^®^ F127, 0.36 g of formaldehyde solution, and 0.36 mL EOA were added in that order into the solvent containing 1.2 g of water, 3.45 g of ethanol, and 112.5 μL of 5 M HCl under stirring at 45 °C for 35 min, similar to our previous study [14]. Then, 2.7 g of PVA solution was added into the solution and continuously stirred at 45 °C for 30 min. Since the pH of the resulting solution was 3.8, MCF synthesized under this acidic condition was named as MCF-A.

The pH of the solution was further controlled for the purpose of increasing the polymerization rate. MCF precursor solutions with different pH values were prepared. Precursor solutions with pH values of 4.6 and 7.6 were made and called MCF-WA and MCF-B precursor solutions, respectively.

For MCF-WA precursor solution, 0.60 g of phloroglucinol dihydrate, 0.60 g of Pluronic^®^ F127, 0.33 g of formaldehyde solution, and 0.47 mL EOA were added in that order into the solvent containing 1.6 g of water, 2.3 g of ethanol, and 75 μL of 5 M HCl under stirring at 40 °C for 30 min. Then, 4.8 g of 13 wt % PVA solution and 375 μL of ammonia solution were further added and stirred at 40 °C for 30 min.

For MCF-B precursor solution, 0.58 g of resorcinol, 0.80 g of Pluronic^®^ F127, 0.64 g of formaldehyde solution, and 0.47 mL EOA were added in that order into the solvent containing 1.7 g of water, 2.3 g of ethanol, and 187.5 μL of 1 M NaOH under stirring at 50 °C for 80 min. Then, 2.4 g of 13 wt % PVA solution was further added and stirred at 60 °C for 60 min. Here, resorcinol and phloroglucinol dihydrate have similar characteristics, but since the polymerization process is slow in an acidic solution, phloroglucinol dihydrate was used rather than resorcinol in order to increase the polymerization rate of MCF precursors [38].

### 3.3. ESD Process of MCF Precursor Solutions

ESD of MCF precursor solutions was processed in the following manner. The precursor solution was placed in a disposable syringe (8 mL). One end of a silicon rubber tube with an internal diameter of 0.3 cm was attached to the tip of the syringe, and the other end of the silicon tube was connected to a 27G (diameter: 0.25 mm) needle by a metal adaptor. The needle was set to an ESD machine (HVU 30P100 made by MECC CO., LTD. (Ogori, Japan)) holder, with a vertical arrangement to the grounded aluminum foil by a distance of 20 cm. The foil stayed on a hot plate at 45 °C, enhancing evaporation of solvents and polymerization of fibers during the ESD process. The humidity was controlled to below RH 40%. The precursor solution in the syringe further filled up the silicon tube and needle by pushing the plunger of the syringe. Finally, MCF precursor fibers were spun by applying a potential difference of +20 to +25 kV under the flow of the precursor solution at 2 mL/h, which was controlled by a syringe pump (KDS 100 made by KD Scientific Inc. (Holliston, MA, USA)). The resulting as-spun fibers were stabilized at 50 °C for 3 h, at 110 °C for 5 h, and then 170 °C for 5 h in the air within the oven.

### 3.4. Calcination Process of MCF Precursors

As a standard heat treatment, three different stabilized as-spun fibers—as-spun MCF-A, as-spun MCF-WA, and as-spun MCF-B—were calcined through a similar heat treatment process as that of MC bulk [14]. Typically, they were heated up to 400 °C with a rate of 2 °C/min, kept at 400 °C for 3 h, further heated up to 800 °C with a rate of 2 °C/min, and kept for 6 h under N_2_ atmosphere, but the heating condition was slightly varied in order to see the effect of heating processes on the mesoporosity and graphitization degree of MCFs. For MCF-A with the 400 °C + 700 °C treatment, the temperature was increased to 700 °C from 400 °C with a heating rate of 2 °C/min and kept for 3 h. For MCF-A with the 400 °C + 700 °C + 800 °C treatment, additional heat treatment up to 800 °C from room temperature with a heating rate of 1 °C/min and 3 h holding was done to MCF-A with 400 °C + 700 °C treatment. For MCF-A with the 400 °C + 700 °C + 800 °C + 900 °C treatment, additional heat treatment up to 900 °C from room temperature with a heating rate of 1 °C/min and 3 h holding was done to MCF-A with 400 °C + 700 °C + 800 °C treatment.

### 3.5. Material Characterization

N_2_ sorption measurements were performed at −197 °C (77 K) by BELSORP-mini (MictrotracBEL Corp. (Osaka, Japan)) after degassing samples at 200 °C under vacuum (below 10^–2^ Pa) for 3 h. XRD patterns were recorded at a scan rate of 2θ = 0.2°/min using a Rigaku SmartLab diffractometer operating at 3 kW (30 mA; 40 kV) with Cu-Ka radiation. CHN elemental analysis was performed using Yanaco CHN Corder MT-5, MT-6 (Yanagimoto Mfg. CO., LTD. (Osaka, Japan)). FESEM observation was performed using a SU-9000 (Hitachi High-Tech (Tokyo, Japan)) by drop-casting sample suspensions onto a carbon-coated copper grid. Nanostructures of MCFs were further characterized using TEM (JEM-2100F; JEOL Ltd. (Tokyo, Japan)). The average pore size was analyzed with selected 120 pores in the TEM image though the image processing.

### 3.6. Electrochemical Characterization

Fundamental electrochemical characterization was performed with a common half-cell setup in an acidic electrolyte. A potentiostat, HZ-7000 (Hokuto Denko (Tokyo, Japan)), was used. All MCFs samples were ball-milled into powder at 800 rpm and prepared as ink suspensions, where 6 μg of MCFs were dispersed into 3 mL of 2-propanol. The working electrodes were prepared by drop-coating 10 μL of the ink suspension onto glassy carbon disks (φ 5 mm). The amount of carbon samples on the glassy carbon disk was kept to 20 μg/cm^2^. The working electrode and a platinum wire used as a counter electrode were placed into 0.1 M HClO_4_ under N_2_ atmosphere at room temperature. Ag|AgCl in a saturated KCl solution was utilized as a reference electrode, and the obtained voltage was converted to the voltage against RHE. Then, CVs were obtained.

Since the thickness of electrochemical double layers in CVs, which represents the amount of stored charge (capacitance), is known to be highly related to the surface area of carbon materials, especially in the research field of supercapacitance [32], specific capacitance C_p_ (F/g) was calculated in this study. Specific capacitance C_p_ can be calculated by the following Equation (1):

(1) $$C\;\left(F/g\right)=\frac{Q_{st}\left(C\right)}{\Delta V\;\left(V\right)\times total\;C\;loading\;\left(g\right)\;}$$

where Δ*V* and *Q_st_* are the voltage window and corresponding double-layer charge, respectively.

CDC measurement is also a common technique to evaluate the capacitance performance of carbon materials. In this study, CDC measurements were done under the N_2_ saturated electrolyte. The charging current was set to 5, 2.5, 1.25, and 0.5 A/g. Then, discharge-specific capacitance C_des_ can be calculated from the discharge curves by the following Equation (2):

(2) $$C=\frac{I\Delta t}{m\Delta V}$$

where *I* (A), *m* (g), Δ*V* (V), and Δ*t* (s) are the discharge current, total carbon loading, potential range, and discharge time consumed in the potential window, respectively.

### 3.7. Evaluation of the Resistance toward Compression

MCF-A was ball-milled at 450 rpm and then mixed with 5 wt % Nafion^®^ solution and ethanol. Typically, 0.105 g of MCF-A was mixed with 0.878 mL of 5 wt % Nafion^®^ solution and 4.800 mL of ethanol. Then, the resulting suspension was spray-printed on the Nafion 212 membrane as a 1 cm^2^ layer, where the carbon loading was 1.5 mg/cm^2^. Finally, it was placed into a cell holder used for regular current–voltage performance measurements after being hot-pressed at 0.3 kN and 135 °C for 190 s. The resistance to compression of 2 Nm for 1 h under this cell holder was evaluated. The cross section of the MCF-A layer was fabricated and observed before and after the compression using FIB-SEM, Helios NanoLab 600i (FEI). When the thickness of the layer was analyzed, images with the smooth layer were selected.

### 3.8. Evaluation of the Through-Plane Electrical Resistance

MCF-As ball-milled at 450 and 800 rpm were spray-printed with a similar method as explained above, but the suspension was instead spray-printed onto 1 × 1 cm^2^ GDL 25BC carbon paper. A sheet of TP1-060T carbon paper was sandwiched between two pieces of GDL 25BC carbon paper coated with carbon layers, and they were assembled into a cell holder as described above. Then, impedance measurements were performed at 0 V from 10 kHz to 0.1 Hz using a PGSTAT 128N potentiostat/galvanostat (Metrohm Autolab (Utrecht, The Netherlands)). Here, an assembly of a sheet of TP1-060T sandwiched between two sheets of GDL 25BC was also evaluated as the baseline for the comparison.

## 4. Conclusions

We successfully synthesized MCFs with controllable pores by combining organic–organic self-assembly and ESD, and optimizing the heat treatment conditions. By changing the pH of the precursor solutions, MCF-A made in the most acidic condition resulted in the largest pore diameter, 4–5 nm among MCFs, and its heat treatment condition was further optimized by stepwise heat treatments of 400 °C + 700 °C + 800 °C in order to get highest carbonization degree, since both pore size and carbonization degree are important for electrocatalyst support. Then, in anticipation of applications in energy devices, several physical properties of MCF-A, including BET specific surface area, electrochemical double-layer capacitance, resistance of compression, and through-plane electronic resistance, were evaluated. As a result, we found that the specific capacitance of MCF-A (56.5 F/g) was 18% higher and 260% higher than conventional XC-72 (47.9 F/g) and CNF (15.6 F/g), respectively. For the resistance to compression, MCF-A layer showed higher resistance (5.5% change) against bulk MC layer (12.8% change). The through-plan resistance was lower when the fiber structure remained more within the thin layer, for example, +8 mΩ for 450 rpm milled MCF-A layer, and +12 mΩ for 800 rpm milled MCF-A layer. These results show that a fiber structure with the high surface area of MCF-A is important as an electrode material. Additional advantages of MCF-A compared with bulk MC suggest that MCF-A has the potential to be used as catalyst supports within electrodes in energy devices.

## Figures and Tables

**Table 1:** Summary of physical properties of MC fibers (MCFs) and bulk mesoporous carbon (MC).

	Pore Diameter (nm)	Surface Area (m^2^/g)	Notes
MCF-A	4.5	370	pH = 2.8
MCF-WA	3.8	340	pH = 4.6
MCF-B	2	670	pH = 7.6
Bulk MC	7.5	635	

**Table 2:** CHN element analysis of MCF-As with the heat treatment up to different temperatures.

Highest Temperature in the Heat Treatment	H Atom (%)	C Atom (%)
400 °C	4.03	76.63
700 °C	1.21	81.24
800 °C	0.91	86.80
900 °C	0.76	86.16

## Data Availability

Not applicable.

## Sample Availability

Samples of the compounds are not available from the authors.
